# Socioeconomic Influences on Outcomes Following Congenital Heart Disease Surgery

**DOI:** 10.1007/s00246-024-03451-7

**Published:** 2024-03-12

**Authors:** Kristin Schneider, Sarah de Loizaga, Andrew F. Beck, David L. S. Morales, JangDong Seo, Allison Divanovic

**Affiliations:** 1 The Heart Institute, Cincinnati Children’s Hospital Medical Center, 3333 Burnet Avenue, MLC 2003, Cincinnati, OH 45229 USA; 2 Department of Pediatrics, University of Cincinnati College of Medicine, Cincinnati, OH USA; 3 Divisions of General & Community Pediatrics and Hospital Medicine, Cincinnati Children’s Hospital Medical Center, Cincinnati, OH USA

**Keywords:** Socioeconomic status, Congenital heart disease, Surgery

## Abstract

Associations between social determinants of health (SDOH) and adverse outcomes for children with congenital heart disease (CHD) are starting to be recognized; however, such links remain understudied. We examined the relationship between community-level material deprivation on mortality, readmission, and length of stay (LOS) for children undergoing surgery for CHD. We performed a retrospective cohort study of patients who underwent cardiac surgery at our institution from 2015 to 2018. A community-level deprivation index (DI), a marker of community material deprivation, was generated to contextualize the lived experience of children with CHD. Generalized mixed-effects models were used to assess links between the DI and outcomes of mortality, readmission, and LOS following cardiac surgery. The DI and components were scaled to provide mean differences for a one standard deviation (SD) increase in deprivation. We identified 1,187 unique patients with surgical admissions. The median LOS was 11 days, with an overall mortality rate of 4.6% and readmission rate of 7.6%. The DI ranged from 0.08 to 0.85 with a mean of 0.37 (SD 0.12). The DI was associated with increased LOS for patients with more complex heart disease (STAT 3, 4, and 5), which persisted after adjusting for factors that could prolong LOS (all *p* < 0.05). The DI approached but did not meet a significant association with mortality (*p* = 0.0528); it was not associated with readmission (*p* = 0.36). Community-level deprivation is associated with increased LOS for patients undergoing cardiac surgery. Future work to identify the specific health-related social needs contributing to LOS and identify targets for intervention is needed.

## Introduction

Pediatric cardiology and cardiothoracic surgery have seen advances in medical and surgical care for patients with congenital heart disease (CHD) that have improved outcomes such that there are now more adults living with CHD than children [1]. Developments in non-invasive imaging have allowed for the expansion of virtual and augmented reality, transforming surgical planning for increasingly complex surgeries, and improvements in mechanical circulatory support, such as ventricular assist device treatment, have broadened options beyond transplantation for patients with heart failure [2, 3]. However, despite these advancements, post-operative mortality remains high and long-term outcomes suboptimal [4]. There have been many investigations into risk factors for adverse outcomes. Factors such as cardiac anatomy, the need for cardiopulmonary resuscitation or extracorporeal membrane oxygenation (ECMO), and prematurity have well-known impacts on surgical outcomes [5]. Identification of such risk factors has led to implementation of strategies and programs such as cardiac arrest prevention plans in intensive care units [6] and home monitoring programs for high-risk patients that have improved morbidity and mortality [7]. While there have been improvements, there is continued room to optimize outcomes. As a result, studies have continued to investigate factors influencing morbidity and survival. More recently, social determinants of health (SDOH)—the context in which children live, grow, and age—have emerged as potentially impactful on outcomes across multiple conditions including childhood asthma and prematurity [8, 9]. Factors such as parental education, insurance coverage, and poverty are elements that may contribute to adverse post-operative outcomes in children with CHD [10, 11].

Previous work has identified the association of SDOH with mortality for children undergoing surgical repair of CHD [12, 13]. Less is known about the impact of SDOH on morbidity, such as length of stay (LOS) and readmissions, in children with CHD. Previous studies have also been limited by narrow outcome assessments or limited measures of SDOH [14, 15]. To help guide strategies to target interventions and improve outcomes for an already vulnerable population, it is important to further investigate and add to the emerging literature of how SDOH contribute to the overall prognosis and trajectory for a child undergoing surgery for CHD. Specifically, we used the deprivation index (DI) to evaluate the impact of community-level markers of SDOH on operative mortality and morbidity, including LOS and 30-day readmission, for patients with CHD requiring surgical intervention.

## Methods

We performed a single-center retrospective cohort study of patients between the ages of 0 and 18 years of age from our institution who underwent cardiac surgery between 2015 and 2018. Patients were excluded if they were missing outcome data or key surgical data, such as STAT category. STAT category, as defined by the Society of Thoracic Surgeons, was used as a marker of procedural risk [16, 17]. Additional data fields collected included birth weight and gestational age, the presence of a genetic syndrome or chromosomal anomaly, age at admission for the surgical procedure, LOS of the hospitalization that included the surgical procedure(s) (in days), readmission within 30 days of discharge, and zip code. Surgical mortality was defined as death occurring within the hospitalization during which the index surgical procedure was performed or death after discharge from the hospital before the end of the 30th post-operative day.

**Fig. 1:**
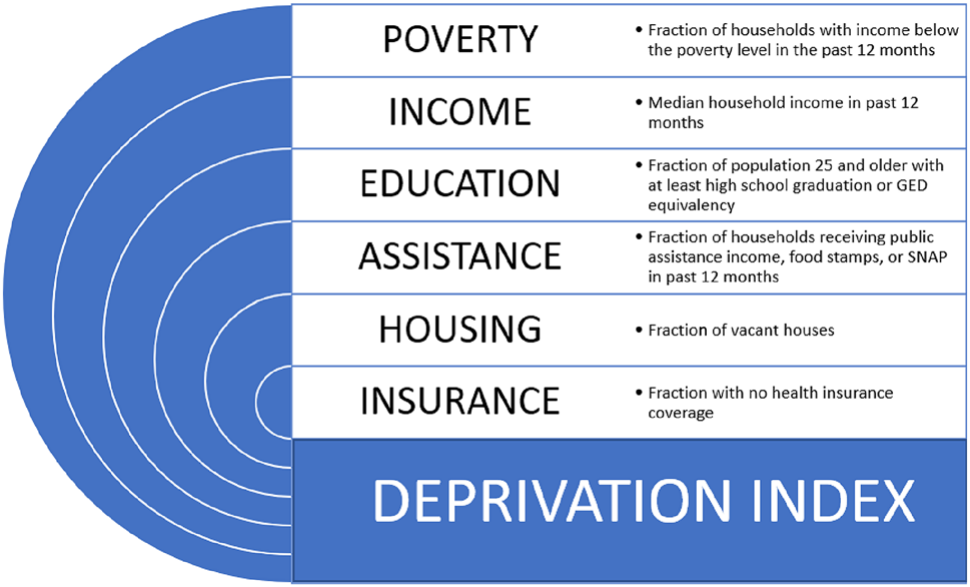
The six variables collected from the American Community Survey that are used to calculate a patient’s Deprivation Index (DI)

A community-level measure of SDOH, a deprivation index (DI), was our primary predictor. The DI was measured at the zip code level to encapsulate community social and economic context and approximate individual-level SDOH [18, 19]. The zip code for each subject was geocoded to a US Census Bureau defined zip code tabulation area (ZCTA). ZCTAs are areal representations of the United States Postal Service zip code service areas. This ZCTA was then used in conjunction with data from the 2010 US Census’ American Community Survey (ACS) to generate a DI for each patient. The DI incorporates six variables associated with material deprivation and ranges from zero to one, with one reflecting the greatest level of community material deprivation (Fig. 1). These variables include poverty, income, education, assistance, housing, and insurance; a detailed description of the variables can be found in Fig. 1. Geocoding and DI derivation were completed at our institution using DeGAUSS [18]. Patient demographic and clinical data were analyzed using descriptive statistics, including medians, interquartile ranges, and/or frequencies reflected as a percentage of the total population. Univariate analysis, general linear mixed-effects models, and generalized mixed-effect regression models were used to determine an association of the DI with mortality, 30-day readmission, and LOS. Due to the rare nature of the events of operative mortality and 30-day readmission, STAT categories 1 and 2 and STAT categories 4 and 5 were grouped together for analyses. Patients in STAT 3 were excluded from these analyses. A *p* value < 0.05 was considered statistically significant. The DI was scaled to provide odds ratios (OR) and mean differences for one standard deviation (SD) increase or decrease in the DI. All analyses were performed using SAS software, Version 9.4 (SAS Institute, Cary NC).

## Results

We identified 1187 patients who met our inclusion criteria. The distribution of the number of patients within each STAT category is shown in Table 1. The DI, birth weight, and gestational age were similar across STAT categories. As expected, given the adjustment for age within the STAT category stratification system, patient age decreased with increasing STAT category. The youngest median age at surgical admission occurred in STAT 4 and STAT 5 patients. The incidence of a major syndrome or chromosomal anomaly was lowest in STAT 5 patients (14.8%) and highest in STAT 3 patients (54.7%).

**Table 1:** Descriptive characteristics of the entire cohort and of the individual STAT categories

Cohort characteristics							
	Entire Cohort	STAT 1	STAT 2	STAT 3	STAT 4	STAT 5	*p* value
Total number of surgical admissions (*n*)	1187	224	433	148	301	81	–
Deprivation index (mean, SD)	0.37 ± 0.12	0.37 ± 0.11	0.37 ± 0.12	0.36 ± 0.13	0.39 ± 12	0.38 ± 0.11	0.18
Age at Admission, years (median, IQR)	0.4 (0, 3.1)	0.9 (0.3, 5.5)	0.6 (0.3, 3.7)	0.5 (0.1, 1.8)	0 (0, 0.7)	0 (0, 0)	< .0001
Birth weight, kilograms (median, IQR)	3 (2.5, 3.4)	3 (2.5, 3.4)	3 (2.5, 3.4)	3.1 (2.6, 3.5)	3 (2.5, 3.5)	3.1 (2.7, 3.4)	0.44
Gestational Age, weeks (median, IQR)	38 (37, 39)	38 (36, 39)	38 (37, 39)	38 (37. 39)	38 (37, 39)	38 (37, 39)	0.33
Syndrome or Chromosomal Anomaly Present (*n*, %)	418 (35.2)	67 (29.9)	137 (31.6)	81 (54.7)	121 (40.2)	12 (14.8)	< .0001
Length of Stay, days (median, IQR)	11 (5, 26)	4 (6, 9)	8 (13, 17)	10 (15, 20)	20 (31, 43)	35 (51, 77)	*

*p values for the length of stay can be found in Table 2

*SD* standard deviation, *IQR* interquartile range

**Fig. 2:**
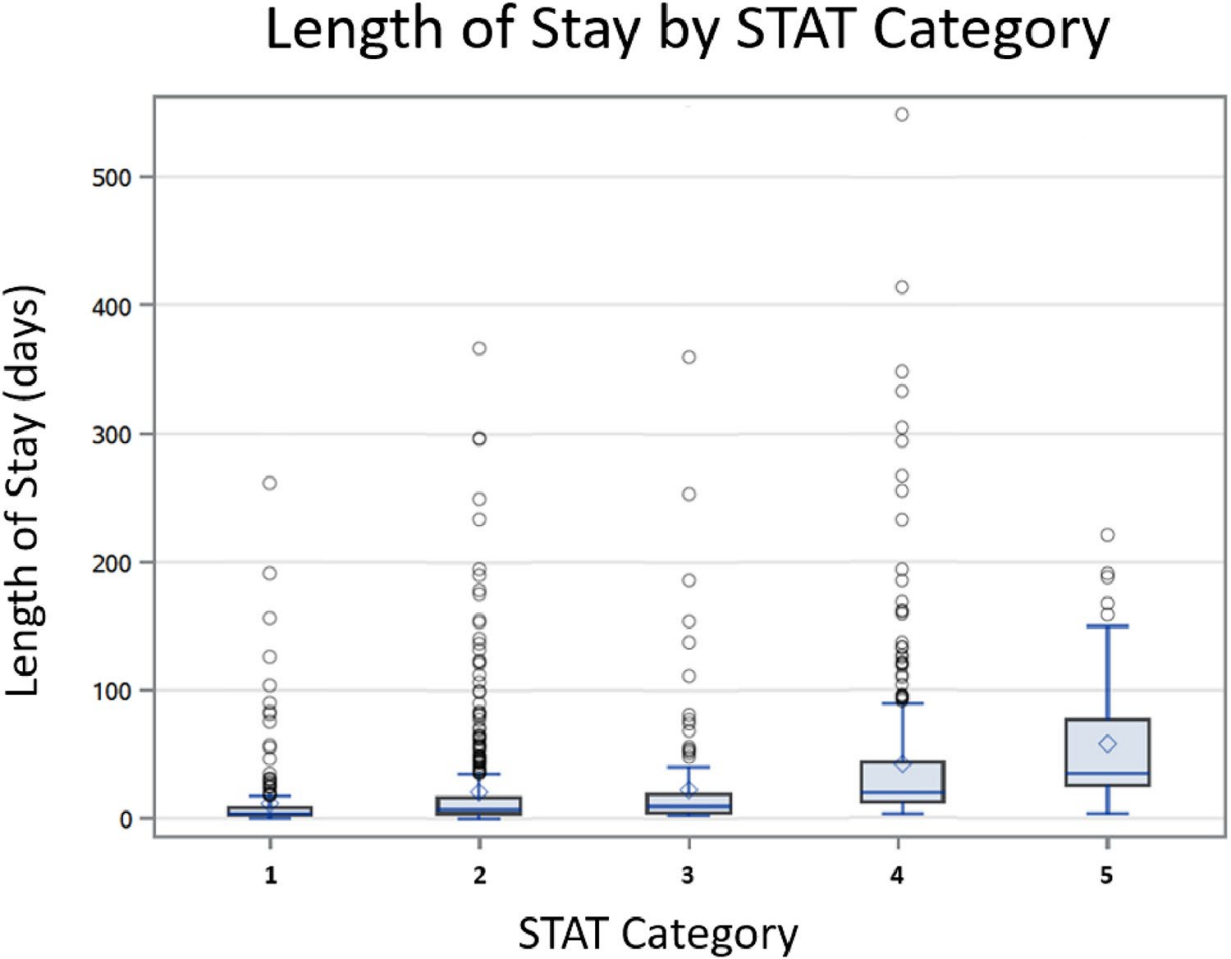
Distribution of length of stay across STAT categories

**Fig. 3:**
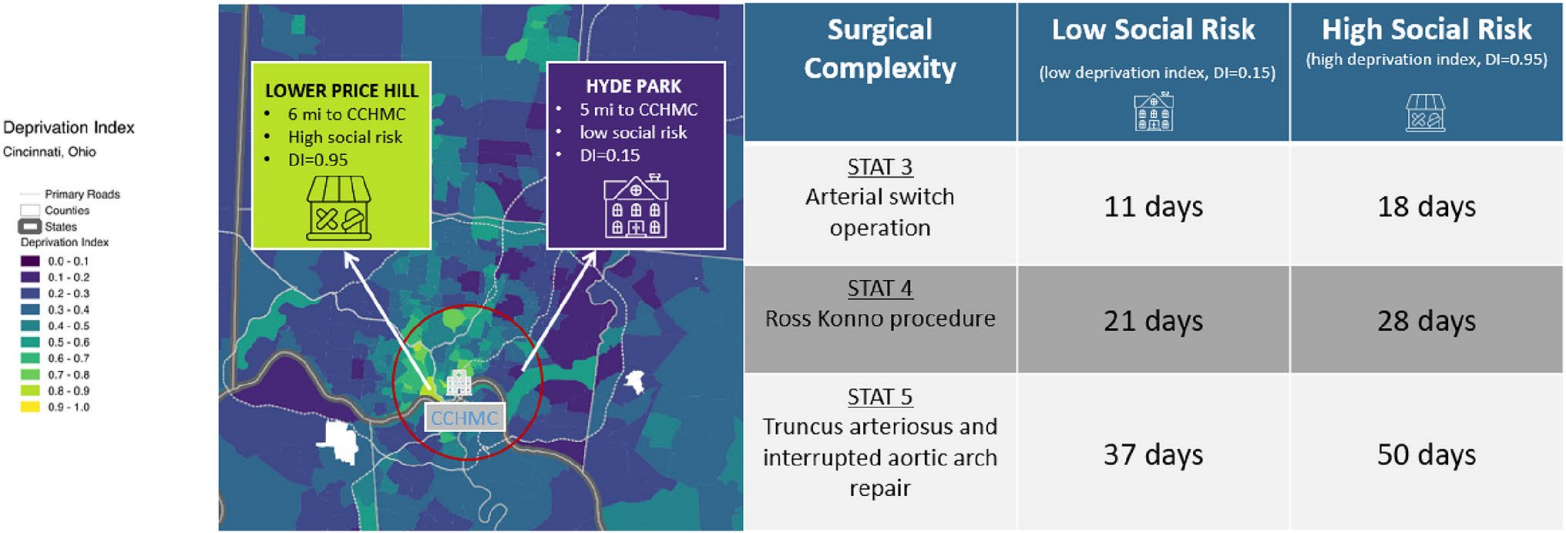
Projected change in length of stay for hypothetical patients in STAT categories 3, 4, and 5 with lower community deprivation and social risk (a low DI) and higher community deprivation and social risk (a high DI). Abbreviations: deprivation index (DI)

The median LOS for the entire cohort was 11 days (IQR 5, 26 days). The median LOS increased with increasing STAT category (Table 1). The shortest median LOS was in STAT 1 patients (4 days, [IQR 6, 9]) and the longest LOS in STAT 5 patients (35 days [IQR 51, 71]). The distribution of LOS across STAT categories is shown in Fig. 2. There was a positive correlation with a higher DI (families living in ZCTAs with more community deprivation) and a longer LOS for those in STAT 3, 4, and 5 categories after adjusting for factors that could contribute to longer LOS such as birth weight, age at admission, and the presence of a syndrome or chromosomal anomaly (*p* < 0.0001) (Table 2). In these patients, with a one standard deviation increase in the DI, LOS was prolonged by more than 5 days in each STAT category with the largest increase in patients undergoing STAT 5 surgeries. Using linear mixed-effect models, examples of the projected change in the LOS with changes in the DI for hypothetical patients in STAT categories 3, 4, and 5 are provided in Fig. 3 [13].

**Table 2:** Change in the length of stay for the entire cohort and by STAT category

	Unadjusted		Adjusted	
	Change in LOS with one SD increase in the DI, days [Beta (SE)]	*p* value	Change in LOS with one SD increase in the DI, days [Beta (SE)]	*p* value
Entire Cohort	6.8 (1.4)	< .0001	5.3 (1.3)	< .0001
STAT 1	0.3 (1.9)	0.87	-0.9 (1.9)	0.64
STAT 2	4.8 (2)	0.0167	2.5 (1.9)	0.2
STAT 3	8.5 (3.1)	0.0075	9 (3.3)	0.0065
STAT 4	9 (3.7)	0.0148	8.8 (3.7)	0.017
STAT 5	13.3 (6.1)	0.0329	15.7 (6.4)	0.016

*LOS* length of stay, *SE* standard error, *DI* deprivation index

The overall mortality for our cohort was 4.6% (55 patients) (Table 3), of which 1% of the patients (12 patients) were STAT category 1 or 2 and 3.4% of the patients (40 patients) were in STAT 4 or STAT 5. There were three STAT 3 mortalities (0.2% of the cohort). The DI approached, but did not meet, a significant association with mortality (*p* = 0.0528). The median DI for patients who died was 0.41 (IQR 0.29, 0.52) compared to a median DI of 0.37 (IQR 0.25, 0.49) for patients who did not.

**Table 3:** Mortality for the entire cohort and by STAT category groupings. Comparisons were not performed among the STAT category groupings given the lack of significance for the overall cohort

	Mortality			
	Number of events	DI for patients with mortality (median, IQR)	DI for patients without mortality (median, IQR)	*p* value
Entire Cohort	55	0.41 (0.29, 0.52)	0.37 (0.25, 0.49)	0.0528
STAT 1 & 2	12	0.40 (0.29, 0.5)	0.35 (0.28, 0.45)	–
STAT 3	3	–	–	–
STAT 4 & 5	40	0.43 (0.32, 0.54)	0.37 (0.29, 0.45)	–

The unadjusted LOS and the LOS adjusted for birth weight, syndrome or chromosomal anomaly, and age at admission are shown

*IQR* interquartile *range*, *DI* deprivation index

There were 91 unique patient readmissions within 30 days of discharge, comprising 7.6% of the entire cohort. There were no significant differences in the DI for readmitted patients (0.38 [IQR 0.26, 0.51]) and those who were not readmitted (0.37 [IQR 0.25, 0.49]) (*p* = 0.36). Forty-five of these events (49% of total readmissions) occurred in the combined STAT 1 and STAT 2 group. There were 33 readmissions (36% of total readmissions) in the combined STAT 4 and STAT 5 group. The STAT 4 and STAT 5 readmissions had a median DI slightly higher (0.39, [IQR 0.28, 0.50]) than the group who had no-readmissions but were not more likely to be readmitted based on their DI (*p* = 0.85).

## Discussion

We found that increased community-level material deprivation, a measure of the SDOH, was associated with a longer length of stay following congenital heart surgery across a wide variety of cardiac surgeries, but not with mortality or readmission at our center. While prior works have demonstrated increased LOS for certain high-risk patient groups or specific surgeries, we found that even in patients undergoing less complex (i.e., lower STAT category) cardiac surgeries, increased community-level deprivation was associated with longer LOS.

The association of SDOH with LOS has been mixed across recent studies, such as those by Bucholz and Spigel. Bucholz et al. demonstrated that a marker of socioeconomic status was not associated with LOS following the Norwood procedure (a STAT 5 procedure) [20]; however, for a similar patient population (patients post Norwood procedure), Spigel et al. demonstrated a significant increase in LOS [21]. While there is variability in the relationship between SDOH and various clinical outcomes, a growing body of literature is demonstrating a relationship between SDOH and increased LOS, including in patients with surgical CHD across multiple STAT categories [22]. The specific underlying factors driving this relationship are not yet clear. Longer LOS is expected with more complex (i.e., higher STAT category) operations with anticipated longer recovery times and often more involved discharge coordination needs. Patients with more straightforward procedures (i.e., STAT 1 operations) typically recover more quickly and have simpler medication regimens and follow-up needs at discharge. Families with greater social needs may be able to navigate a shorter and less complex admission associated with lower risk cardiac surgeries, but the prolonged strain of an admission for higher risk surgical procedures may be overly taxing to their resources. Patients who have higher risk procedures or longer lengths of stay may have additional non-cardiology services that are needed after discharge such as physical, occupational, or speech therapy to build or regain skills that were delayed during the prolonged admission. In these patients, there is a greater requirement of families to navigate maintaining life’s demands during their child’s admission, understanding their child’s more complex medical requirements, medication regimens, and more intense outpatient care needs.

Patient discharges are delayed not only due to medical requirements, but also due to family obligations outside the hospital. These may include caregiver limitations to be present for discharge education due to their work schedules, other childcare considerations, or transportation issues. Additionally, certain patients may remain inpatient longer while strategies to minimize the risk of readmission and plans to ensure that the family will be able to follow-up for outpatient post-operative care are put in place. This often entails working with a multidisciplinary team of social workers, dieticians, pharmacists, and inpatient and outpatient nurses and cardiologists to address barriers to optimal discharge readiness, such as appropriate post-discharge supplies and equipment, or transportation to the outpatient follow-up appointment. Patients in higher STAT categories typically have more complex care needs, requiring additional coordination and investment to ensure a safe discharge and minimize the likelihood of readmission. Navigating this sometimes-taxing process may be intensified for those caregivers who face these challenges amidst negative SDOH, likely with fewer resources to absorb the significant financial and temporal investment required to care for post-operative children with complex CHD. At our institution, significant effort goes into ensuring all post-operative patients and families are medically and socially ready for discharge, even if it results in longer LOS; this may contribute to our finding that community-level deprivation was not associated with readmission in our cohort.

Prolonged LOS following CHD surgery is associated with worse patient outcomes and incurs high personal and financial stress to the family. Longer LOS after congenital heart surgery has been shown to be associated with increased morbidity, such as increased risk of hospital acquired infections [23, 24] and worse neurodevelopmental outcomes [25]. Because of the association of shorter LOS with improved patient outcomes, some advocate to include LOS as one of the quality metrics for congenital heart surgery [26]. However, factors that affect a metric like LOS are numerous and complex, with growing evidence that SDOH impact LOS independently of medical and surgical complexity. Hence, work to identify metrics and strategies that could lead to better outcomes for patients with CHD must be careful not to create unintended disparities. By accounting for SDOH and being mindful of inequity gaps, improvement interventions are more likely to improve outcomes, such as LOS, without unintentionally widening outcome differences. Further work is needed to investigate what specific components of SDOH and health-related social needs drive longer LOS and may serve as targets for future interventions to equitably decrease LOS and improve outcomes. Potential areas for improvement could include an automatic visit with a social worker for all pre-surgical patients (rather than only those who self-refer or are referred by their cardiologist) to do a detailed needs assessment and ensure common needs, such as lodging and transportation, have been addressed beyond simply getting to the hospital the day of surgery and home the day of discharge. Other programs for patients with a prenatal diagnosis could focus on maternal nutrition to positively impact both maternal and fetal outcomes. Interventions to address specific SDOH components will also need to be flexible to adapt to individual patient and family needs.

The mortality rate in our cohort was similar to the reported Society of Thoracic Surgeons mortality for the individual STAT categories during the time period of the study [4]. Prior works have shown an association between mortality and SDOH [20, 27]. Anderson et al. found that children from lower-income neighborhoods undergoing cardiac surgery had increased odds of mortality [14]. Bucholz et al. and de Loizaga et al. used census-based neighborhood scores or indices to examine neonates with single ventricle heart disease, and both demonstrated an association of SDOH markers and mortality. In our study, the DI approached but did not meet a significant association with mortality. Our results may have been influenced by sample size as mortality is a rare event, which may have made it difficult to gather sufficient patients to detect an effect in a single-center study. Unlike our study, Anderson, de Loizaga, and Bucholz were database studies with larger cohorts.

Our study has several limitations. While our single-center cohort was not small, larger multi-center or registry studies will be needed to further analyze the relationship between mortality and SDOH. There are current efforts among national registries such as the Pediatric Acute Care Cardiology Collaborative (PAC3) to examine these outcomes across multiple institutions and will hopefully provide insights into these associations [28, 29]. As with mortality, the factors that drive LOS are complex. We chose to model a linear relationship between community deprivation and LOS, but clinically there are multiple factors that influence a patient’s discharge date, and the relationship is likely more complex. We attempted to account for some of these considerations by adjusting for factors known to affect LOS, such as birth weight, age at admission, and the presence of a syndrome or chromosomal anomaly, but given the myriad of factors than can affect LOS, the adjustment is likely incomplete. Our LOS metric was captured in such a way that it did not differentiate pre- and post-operative length of stay. The LOS for patients who do not undergo surgery on the day of admission, such as neonates with prenatally diagnosed congenital heart disease, will thus be longer. We attempted to control factors that could significantly prolong the pre-operative length of stay as discussed above, but further work will be needed to investigate the impacts of socioeconomic factors on post-operative length of stay. Furthermore, the DI is generated using a patient’s permanent address and does not account for alternative discharge destinations such as to long-term care facilities or short-term bridges to home near the hospital, like the Ronald McDonald House. While these discharge plans may offer closer access to medical resources following discharge and help mitigate certain health-related social needs (such as transportation barriers), this benefit may be outweighed by effect on other needs (continued financial burden and isolation from social supports). Future work with larger data sets, analysis of the myriad of components that make up socioeconomic deprivation, and examining the impact of different discharge practices should be considered to provide greater clarity on this complex topic. Additionally, the DI is a community-level measure and therefore may not reflect the individual’s risk. Moreover, although census tracts are more specific to assess community-level deprivation, we were limited to the more heterogeneous zip codes or ZCTAs, potentially further biasing our results toward the null. Finally, as with any retrospective study there is the potential for missing data and selection bias.

## Conclusion

We found that social determinants affect health outcomes in patients with a wide range of cardiac surgical disease in the form of longer post-operative lengths of stay. Those undergoing more complex cardiac surgeries were most affected. Our findings add to the growing work recognizing the significance of SDOH and outcomes for children with CHD, highlighting the need for ongoing research into the specific drivers of these findings and thus potential targets for interventions.
